# Prevalence, Associated Factors and Psychological Determinants of Obesity among Adults in Selangor, Malaysia

**DOI:** 10.3390/ijerph18030868

**Published:** 2021-01-20

**Authors:** Sherina Mohd-Sidik, Rampal Lekhraj, Chai Nien Foo

**Affiliations:** 1 Department of Psychiatry, Faculty of Medicine and Health Sciences, Universiti Putra Malaysia, Serdang 43400, Malaysia; 2 Department of Community Health, Faculty of Medicine and Health Sciences, Universiti Putra Malaysia, Serdang 43400, Malaysia; dr_rampal1@hotmail.com; 3 Department of Population Medicine, Faculty of Medicine and Health Sciences, Universiti Tunku Abdul Rahman, Kajang 43000, Malaysia

**Keywords:** obesity, risk factors, psychological, adults, Malaysia

## Abstract

Background: The pervasiveness of obesity is a growing concern in the world. This study aims to determine the prevalence of obesity among a segment of the Malaysian population, as well as investigate associated factors and psychological determinants of obesity. Methods: A cross-sectional study design was carried out in Selangor, Malaysia. A total of 1380 Malaysian adults (≥18 years old) participated in a structured and validated questionnaire survey. TANITA body scale and SECA 206 body meter were used to measure the respondents’ weight and height, from which measurements of their body mass index (BMI) were calculated. Results: The overall prevalence of obesity (BMI ≥ 30 kg/m^2^) among adults in Selangor, Malaysia, was 18.6%. Factors significantly associated with increased risk of obesity were: being female (OR = 1.61, 95% CI [1.20–2.17]), aged between 30 to 39 years old (OR = 1.40, 95% CI [1.04–1.88]), being Indian (OR = 1.55, 95% CI [1.13–2.12]), married (OR = 1.37, 95% CI [1.03–1.83]), and having only primary school education (OR = 1.80, 95% CI [1.17–2.78] or secondary school education (OR = 1.37, 95% CI [1.04–1.81]). In the multiple linear regression analysis (stepwise method), perceived stress (B = −0.107, *p* = 0.041), suicidal ideation (B = −2.423, *p* = 0.003), and quality of life in the physical health domain (B = −0.350, *p* = 0.003) inversely and significantly contributed to BMI among males. Among females, stressful life events contributed positively to BMI (B = 0.711, *p* < 0.001, whereas quality of life in the psychological domain had a negative effect (B = −0.478, *p* < 0.001) in this respect. Conclusion: There is an urgent need to integrate psychological approaches to enhance the effectiveness of obesity prevention strategies and weight-loss programs.

## 1. Introduction

Obesity is a major global concern as it increases nations’ clinical burden [1,2,3]. Compared to other Asian countries, the prevalence of obesity in Malaysia topped the list partly due to Malaysians’ eating behaviour [4,5]. Although obesity does not directly impact morbidity and mortality, it increases the risk of various chronic diseases such as diabetes, osteoarthritis, cancers, and major vascular diseases [6,7,8,9]. Mortality from these chronic diseases is highly associated with obesity [10]. Besides, obesity also increases loading at the weight-bearing joint, leading to functional locomotor disability, the main cause of physical inactivity that contributes eventually to increased morbidity and mortality [11,12,13]. In other words, obesity indirectly shortens life expectancy while directly impacting the individual’s quality of life and psychological well-being as it could lead to depression or low self-esteem [14,15,16,17].

Body mass index (BMI) can be a screening tool for weight category and moderately correlated with more direct body fat measures [18]. The World Health Organization (WHO) defines overweight and obese persons as those having BMI greater than or equal to 25 and 30 kg/m^2^, respectively. According to the Global Burden of Disease Study (2013), an estimated 2.1 billion people had a BMI of 25 kg/m^2^ or greater. The study showed that 14.1% of adults were obese (11.4% of men and 16.7% of women) [6]. The combined percentage of overweight and obesity prevalence increased by 28% in adults between 1980 and 2013 [6]. According to the WHO global status report on non-communicable diseases (2014), an estimated 39% of adults were overweight (38% of men and 40% of women), while 13% of adults were obese (11% of men and 15% of women) [19]. The prevalence of being overweight and obese is expected to rise by 2025. However, the prevalence of obesity among the adult population between 2014 and 2016 remained the same at 39% [20].

In many developing countries, including Malaysia, the prevalence of obesity, especially among urban dwellers has reached epidemic levels [21]. Among Southeast Asian countries, Malaysia recorded the highest obesity rate in adults [6,22]. Between 1996 [23] and 2015 [24], the prevalence of obesity among adults in Malaysia showed a four-fold increase, surging from 4.4% to 17.7%. In 2011 and 2015, the prevalence of being overweight among Malaysian adults was 29.4% and 30%, respectively, while during the same period, obesity accounted for 15.1% and 17.7%, respectively [25]. In the National Health and Morbidity Survey (NHMS) 2019, the current prevalence of obesity among Malaysian adults was 19.7% [26]. Pahang, Pulau Pinang, Terengganu, and Sabah showed the lowest rates of obesity (15.7%–18.6%) in comparison to other states in Malaysia [26]. Between 2011 and 2019, there was an alarming rate of increase in obesity among adults in Selangor (17.1% and 19.3%) [26], a highly populated state in Malaysia [25]. As well as geographic patterning, there are ethnic disparities in the prevalence of obesity among Malaysian adults. Obesity was more prevalent in the Indian and Malay communities as compared to other ethnic groups (Chinese and others) [27,28].

Obesity is frequently linked to psychosocial issues such as low self-esteem, depression, anxiety, stress, suicidal ideation, and quality of life [29]. A recent systematic review and meta-analysis study showed that obese adults had up to 32% higher risk of depression than those underweight [30]. A meta-analysis and systematic review by Gariepy, Nitka, and Schmitz revealed a moderate level of evidence for a positive association between obesity and anxiety disorders [31]. Increased BMI is significantly associated with stressful life events and perceived stress. In 2011, a meta-analysis from 14 cohort studies showed a weak positive association between stress levels (general life stress, caregiver stress, work stress) and BMI. The stress level was at its highest among subjects who were morbidly obese (BMI ≥ 40 kg/m^2^) [32]. A cross-sectional study conducted among 5118 Australian adults (≥25 years old) showed that individuals who experienced more than three stressful life events and had high levels of perceived stress, had higher BMI than those who had no stressful life events and had a low level of stress respectively [33]. Similarly, stress has been reported in other research to be associated with obesity [34].

“Common obesity” since some types of obesity are, in fact, a consequence of hormonal abnormalities related to syndromes and genetic disorders, reflects the uncontrollable individual’s decision and engagement in behaviour that sustains weight management. Neurophysiological pathways might influence an individual’s eating behaviour that undermines individuals’ ability to make thoughtful cognitive decisions [35]. This study investigated several factors that were thought to impact the obesity of Malaysians. The authors hypothesized that personal psychological distress (depression, anxiety, self-esteem, and suicidal ideation) and environmental factors (perceived stress, stressful life events, and quality of life) would emerge as significant contributors to obesity.

Given that obesity has been linked with significant health-related conditions and psychological effects, obesity prevention is imperative. Understanding the multiple and complex psychological determinants for obesity is crucial for developing effective prevention efforts. Despite research on the geographic and socio-demographic factors associated with obesity, there is still a paucity of studies on psychological factors leading to obesity in Malaysia. Given the incidence of obesity was amongst the highest in Selangor compared to other states in Malaysia and interactions between psychological factors leading to obesity are not yet well understood, this study aims to ascertain the prevalence, associated factors (socio-demographic variables and the presence of chronic diseases), and psychological determinants of obesity among adults in the state of Selangor, Malaysia.

## 2. Materials and Methods

### 2.1. Study Design and Sampling Frame

A cross-sectional study design was utilised with a 4-stage stratified sampling conducted by the Department of Statistics Malaysia (DOS). In the first stage, three districts (Hulu Langat, Sepang, and Klang) out of nine districts in Selangor were chosen in this study. They had almost 2.2 million people, and this proportion covered almost half of the entire population of Selangor. In the second stage, 157 Enumeration Blocks (EBs) were selected and allocated according to the particular districts’ proportional population size; 84 EBs were selected in Hulu Langat, 60 EBs in Klang, and 13 EBs in Sepang. In the third stage, living quarters (LQs) were selected in each EB using a systematic random sampling method. LQs in this study represented households in the community. Each EB comprised eight LQs. Finally, the respondents were randomly selected from 1256 LQs.

### 2.2. Sample Size

In the sample size calculation for primary outcome variable, the proportion of poor mental health status in Malaysia (11.2%) was obtained from Malaysia’s third NHMS [36]. The absolute error or precision was set at 2% and the response rate was set at 50%. The final estimated sample size determined by Kish L (1965) [37] was 1912 subjects that comprised the major racial groups of Malays, Chinese, and Indians.

### 2.3. Eligibility Criteria

The respondents in this study were Malaysian citizens, 18 years old and above, who were residents in the selected LQs.

### 2.4. Data Collection

Data of eligible respondents were collected during home visits by the principal researcher and a team of trained research assistants, from June to December 2012. Data on socio-demographic (gender, age, ethnicity, urban/rural residence, marital status, education level, employment status), weight, height, medical history, disability, and level of psychological status (depression, anxiety, perceived stress, self-esteem, suicidal ideation, stressful life events, and quality of life) were obtained during the home visits. The dependent variable of this study was BMI. The independent variables were gender, age, ethnicity, urban/rural residence, marital status, education level, employment status, the presence of chronic diseases (heart disease, diabetes, stroke, hypertension, arthritis, cancer, asthma, kidney failure, and thyroidism), depression, anxiety, perceived stress, self-esteem, suicidal ideation, stressful life events, and quality of life. A set of self-administrated, pre-tested, and validated questionnaires (available in English and Malay versions) was used in this study. Malay is Malaysia’s national language, and most Malaysian citizens can read either Malay or English or both. The measures of this study are described below.

#### 2.4.1. Obesity

In this study, obesity was defined as having a BMI of 30 kg/m^2^ or greater [38]. The TANITA weighing scale (accuracy up to 0.1 kg) was used to measure body weight, while the SECA 206 Body meter (accuracy up to 0.5 mm) was used to measure height. One measurement for weight and height was taken of the participant without shoes and in light clothes by trained research assistants with the principal researcher’s monitoring to assure the quality of data collected. The BMI was calculated by using the formula weight (kg)/ height (m^2^).

#### 2.4.2. Depression

The English and Malay versions of the 9-item Patient Health Questionnaire-9 (PHQ-9) [39] were adapted to assess depression in this study. Respondents were asked to rate the extent of depression symptoms experienced over a fortnight. The total scores ranged from 0 to 27, where higher total scores indicated more severe levels of depression. A total score of 10 and above was considered as having depression [40]. PHQ-9 is a reliable and valid instrument to measure depression, with an overall Cronbach’s alpha of 0.89 [39]. The Malay version’s reliability and validity score for PHQ-9 was good, with an overall Cronbach’s alpha of 0.70 [40].

#### 2.4.3. Anxiety

The English and Malay versions of the 7-items Generalized Anxiety Disorder-7 (GAD-7) questionnaire [41] were adapted to assess anxiety among respondents in this study. The respondents were asked to rate each item according to a four-point Likert scale rating from 0 to 3 (0 = “not at all” to 3 = “nearly every day”). The scores ranged from 0 to 21, whereby higher scores indicated higher levels of anxiety. Respondents with a total score of 8 or above were considered as having anxiety [42]. GAD-7 is a reliable and valid instrument to measure anxiety, with an overall Cronbach’s alpha of 0.92 [41]. The Malay version’s reliability and validity score for GAD-7 was good, with an overall Cronbach’s alpha of 0.74 [42].

#### 2.4.4. Perceived Stress

The English and Malay versions of the 10-item Cohen’s Perceived Stress Scale (PSS) questionnaire [43] were adapted to assess the respondents’ perceived stress in the month prior to the survey. The questionnaire was self-administrated, using a five-point Likert scale where the total scores ranged from 0 to 40. A higher score indicated higher perceived stress among the study respondents. PSS is a reliable and valid instrument to measure the perceived stress, with an overall Cronbach’ alpha of 0.86 [43]. The Malay version for PSS was reliable and valid, with an overall Cronbach’s alpha of 0.63 [44].

#### 2.4.5. Self-Esteem

The English and Malay versions of the 10-item Rosenberg’s Self-esteem Scale (RSES) questionnaire [45] were adapted to assess self-esteem in this study. For each item, a four-point Likert scale with ratings 1 to 4 was used. The total scores ranged from 1 to 40, where higher scores indicated higher self-esteem. RSES is a reliable and valid instrument to measure self-esteem, with an overall Cronbach’ alpha of 0.88 [45]. The Malay version’s reliability and validity score for RSES was good, with an overall Cronbach’s alpha of 0.80 [46].

#### 2.4.6. Stressful Life Events

The 17-item Kendler’s Stressful Life Events questionnaire [47] was adapted to assess stressful life events experienced by the respondents in the previous year. For each item, a “yes” scored 1 point and “no” scored 0 point. A total score of 1 point indicated low stressful life events, 2 points indicated moderate stressful life events, and 3 points or above indicated high stressful life events [33].

#### 2.4.7. Suicidal Ideation

Respondents were categorised as having suicidal ideation if they answered “Yes” to the ninth question in the PHQ-9 [39], which was “Thoughts that you would be better off dead or of hurting yourself in some way”.

#### 2.4.8. Quality of Life

The World Health Organization Quality of Life Assessment (WHOQOL-BREF-26) [48] was adapted to assess quality of life in this study. WHOQOL-BREF-26 is a 26-item self-administrated questionnaire which assesses four different domains, viz. (i) physical health, (ii) psychological health, (iii) social relationships, and (iv) environment [49]. Respondents report how much they have experienced the situation described in the questionnaire in the past two weeks on a 5-point Likert scale: 1 = not at all and 5 = completely. Each domain’s mean score was multiplied by 4, where a higher score indicated higher quality of life in that specific domain.

### 2.5. Statistical Analysis

After being screened and verified, the data in this study were analysed using IBM SPSS Statistics Version 23.0. Questionnaires with missing data were excluded from the analyses. Descriptive statistics were used to present categorical data as frequencies and percentages. Mean and standard deviation (SD) were used to describe interval and scale data in this study. Chi square test (χ^2^) was used to assess the association of socio-demographic characteristics and chronic diseases with obesity. Fisher’s exact test (FET) was referred when the expected frequency for a particular category was less than five. The Phi coefficient (2 × 2 cells) and contingency coefficient (other than 2 × 2 cells) were used to measure the strength of association between the dependent variables and independent variables, where <0.2 was negligible association, 0.2–0.4 was low association, 0.4–0.7 was moderate association, 0.7–0.9 was high association, and >0.9 was very high association (Guildford rule of thumb). Odds ratio (OR) in chi-square (2 × 2 cells) was used to measure the relative risk between the two groups in each social demographic characteristic (e.g., ethnic Malay and non-Malay; Chinese and non-Chinese; Indian and non-Indian). Multiple linear regression analysis (stepwise method) was used to assess the relationship between psychological factors and obesity. Variables with a *p* value of less than 0.25 in simple linear regression were included in the multiple linear regression model. The level of significance was set at a *p* value of less than 0.05 with 2-tailed test.

### 2.6. Ethics Statement

This study was approved by the Universiti Putra Malaysia Ethics Committee for research involving human subjects (JKEUPM), prior to the commencement of the study. The study respondents were recruited after obtaining their informed consent (written and verbal).

## 3. Results

Of the 1912 respondents selected, 1556 consented, thus resulting in a response rate of 81.38%. In this study, 1380 respondents were included for analysis after excluding some questionnaires for missing data (inclusion rate of 88.69%).

The majority of the study respondents were female (62.83%), aged between 20 to 29 years old (36.9%), Malays (69.1%), residing in urban areas (93.1%), married (61.7%), had attained secondary education (49.9%), and were employed at the time of data collection (51.6%). A total of 23.6% of the respondents had a history of medical problems. Approximately 1.6% of the respondents had past history of mental health problems, while 2.5% had family members with mental health problems (Table 1).

The respondents had a mean BMI of 25.34 kg/m^2^ (SD = 5.79), self-esteem of 19.65 (SD = 3.68), and perceived stress of 15.08 (SD = 4.77). A total of 7.1% of the respondents had depression. Anxiety and suicidal ideation are prevalent among 5% and 7% of the respondents, respectively. Overall, approximately 73.1% had experienced high stressful life events in the previous year. The overall mean score for quality of life was highest in the social relationship domain at 15.28 (SD = 2.42) compared with 14.79 (SD = 2.16) for the physical health domain, 14.56 (SD = 2.09) for the psychological health domain, and 14.66 (SD = 1.97) for the environment domain (Table 1).

Table 2 shows that the prevalence of obesity among adults in Selangor was 18.6%, with a higher proportion being females (21.1%) compared to males (14.2%). In Table 1, the mean BMI measurements for males and females are almost identical (25.26 vs 25.38), yet more females are obese. Table 2 shows no obvious peaks by age. Males: 19 years = 17%, 20–29 = 16%, 60 = 15%; Females: 30–39 = 28%, 40–49 = 26%, 60 = 26%. The highest prevalence of obesity was found among the Indian respondents (24.2%; 20.7% males and 25.8% females). The proportion of obesity was higher for those residing in urban areas (18.7%), the widowed (26.5%), those who had attained only primary school education (27.8%), as well as the unemployed (20.2%), especially among females compared to males. The differences in distribution of all the study variables between males and females were not statistically significant (Table 2).

In this study, the strength of association between the variables was determined based on the Phi coefficient and contingency coefficient. At the same time, the crude relative risk was obtained from Chi-square test. The findings showed a significant (*χ*^2^ (10.09), *p* = 0.001) but negligible (φ = 0.086) association between obesity and gender. Females were significantly associated with obesity by 1.6 times compared to males (OR (crude) = 1.61, 95% CI [1.20–2.17]). Age had a significant (*χ*^2^ (12.84), *p* = 0.025) but negligible (φ = 0.096) association with obesity. Two age groups were found to have a significant association with obesity. Those who were less than 30 years old had a reduced risk of obesity, especially among those aged 20–29 years old (OR (crude) = 0.64, 95% CI [0.48–0.87]). Respondents aged ≥ 30 years old had an increased risk of being obese, especially those aged 30–39 years (OR (crude) = 1.40, 95% CI [1.04–1.88]). The proportion of obesity was significantly higher among Indians compared to other ethnicities (24.2%; *χ*^2^ (11.03), *p* = 0.012). Being an Indian increased the risk of obesity by 55% (OR (crude) = 1.55, 95% CI [1.13–2.12]) compared to other ethnicities (Table 3).

Marital status showed a significant but negligible association with obesity (*χ*^2^ (18.57), *p* = 0.001). Those who were single were less likely to be obese (OR (crude) = 0.63, 95% CI [0.46–0.85]); however, being married carried a significantly higher risk of obesity (OR (crude) = 1.37, 95% CI [1.03–1.83]). Individuals with primary or secondary education had a significantly higher risk of being obese (OR (crude) = 1.80, 95% CI [1.17–2.78] and OR (crude) = 1.37, 95% CI [1.04–1.81] respectively). On the other hand, those with tertiary education had a lower risk of being obese (OR (crude) = 0.57, 95% CI [0.43–0.77]). The results showed that rural/urban residency (*χ*^2^ (0.197), *p* = 0.657), as well as employment status (*χ^2^*(2.66), *p* = 0.256), were not associated with obesity (Table 3).

Table 4 shows the association between obesity and chronic diseases. Having various chronic diseases (*χ*^2^ (19.00), *p* = 0.001; OR (crude) = 1.92, 95% CI [1.43–2.56]) was significantly associated with obesity, especially diabetes (*χ*^2^ (10.37), *p* = 0.001; OR (crude) = 2.01, 95% CI [1.31–3.10]), hypertension (*χ*^2^ (22.80), *p* = 0.001; OR (crude) = 2.37, 95% CI [1.65–3.41]), asthma (*χ*^2^ (3.99), *p* = 0.046; OR (crude) = 1.71, 95% CI [1.00–2.92]) and thyroidism (*χ*^2^ (5.27), *p* = 0.043; OR (crude) = 4.44, 95% CI [1.10–17.89]).

In the multiple regression analysis, perceived stress (B = −0.107, *p* = 0.041), suicidal ideation (B = −2.423, *p* = 0.003), and quality of life in the physical health domain (B = −0.350, *p* = 0.003) were negatively associated with obesity among males (Table 5). Among females, stressful life events (B = 0.711, *p* < 0.001) contributed significantly to obesity, whereas quality of life in the psychological health domain (B = −0.478 *p* < 0.001) negatively contributed to obesity (Table 6).

## 4. Discussion

Rapid economic growth and social changes (e.g., increasing sedentary lifestyles and the involvement of more women in the workforce) in Malaysia have led to an increase in eat-out practice [50,51], which may have contributed to the increased prevalence of obesity in Malaysia between 2011 and 2015. The results of this study also correspond to the NHMS carried out in 2015, which found that the prevalence of obesity was 18.7% among Malaysian adults in Selangor [24]. Based on our findings, the overall prevalence of obesity among adults in Selangor, Malaysia was 18.6%, consisting of 14.2% of men and 21.1% of women. This was an increased prevalence of obesity (BMI ≥ 30 kg/m^2^) by 3.6% compared to 15.1% in 2011 [52], and rising to 17.7% in 2015 [53]. The prevalence of obesity in China and India (around 2013) was far lower as compared to this current study (4.4% and 4.0%, respectively) [6]. Given the current urbanization trend in Malaysia, with Selangor amongst the more urbanized states, the increase in obesity might be due to changes in the daily diet (e.g., high-calorie food intake and choice of food), more sedentary lifestyle, and metabolic dysfunction [51,54], despite numerous obesity prevention programs being conducted [55,56,57].

In this study, it was found that women were more likely to be obese compared to men. This is consistent with a study in the Southeast Asian region, which found that the number of obese women was almost double that of obese men [19]. Similarly, a study conducted among women in Selangor found that the prevalence of obesity was 16.7% [58]. Although women generally ate slightly healthier food than men, most of them lacked physical activity [59]. Besides, the impact of pregnancy and childbirth could have influenced the development of obesity [54].

The findings in this study were inconsistent with that of another local study in Selangor that showed that the prevalence of obesity was higher among respondents between 40 and 59 years of age in both genders [60]. This current study indicated that the critical timeframe of weight gain in males appeared to be during the post-secondary-education period, mostly when the respondents had just left the structured school lifestyle and had a less structured and less externally-monitored environment such as the workplace or college/university [54]. Besides that, increased consumption of convenient food at the workplace or tertiary institutions while coping with busy schedules might contribute to obesity [61,62,63]. The high prevalence of obesity among women aged between 30 to 49 years old could be due to pregnancy or menopause [54,64].

Consistent with the current findings, a cross-sectional study among 16,127 Malaysians aged ≥ 15 years old found that Indians, followed by Malays, had a higher prevalence of obesity than the Sarawakian indigenous group, the Chinese, and the Sabahan indigenous group [28]. Similarly, a recent study by Wan Mohamud and colleagues found that among 4428 adults aged 18 years old and above, Indians and Malays had the highest prevalence of obesity [27]. Another recent study in 2016 also reported that the prevalence of obesity among Indians in Malaysia was highest, followed by the Malays, while the Chinese were the least obese ethnic group in the country [65]. The metabolic syndrome that disproportionately affects Indians in Malaysia is less engagement in physical activity and the consumption of fewer fruits and vegetables than Malays and Chinese [66].

In this study, obesity was also more prevalent among those who were married and had attained only primary and secondary education; these results were consistent with previous studies evaluating the associations of marital status [67,68] and educational background [69,70] with obesity. Generally, having a lower education background was associated with a lack of access to health-related information and healthy lifestyle choices, leading to higher body weight [71]. Individuals in different social positions have different coping mechanisms, and each of them will experience a different level of perceived stress, which significantly influences the probability of being obese [72].

The detrimental health consequences of obesity have been shown to contribute to three of the four most significant non-communicable diseases (diabetes, cardiovascular disease, cancer, and respiratory disease) [19,73,74]. This is in line with this study’s findings, which showed increased risk associations with diabetes, hypertension, asthma, and thyroidism as BMI reached 30 kg/m^2^ and above. Data from systematic reviews of 97 cohorts among 1.8 million respondents showed that raised blood pressure contributed 31% to coronary heart disease risk among obese people [6]. The current findings are supported by the Malaysia’s NHMS in 2011 where almost 1 in 7 overweight and obese Malaysian adults had type 2 diabetes mellitus [52]. It is important to note that dietary glycemic index (GI) may play an essential role in body weight regulation.

Besides, our findings suggest that contributing psychological factors to BMI between males and females vary slightly. Based on our findings, perceived stress, suicidal ideation, and quality of life in the physical health domain had significant and negative relationships with BMI among males. In this study, suicidal ideation was present among male respondents (9.6%). It is vital to introduce routine screening for suicidal ideation, although the relationship between suicidal ideation and BMI was negative. The physical health domain (quality of life) was a significant predictor of BMI among the male respondents. Consistent with these findings, a study by Truthmann et al. from the German Health Interview and Examination Survey 2008 to 2011 found that BMI was significantly associated with lower physical health quality of life [75]. A systematic review by Warkentin et al. [76] reported that there was an interrelationship between weight loss and modest improvement in physical health quality of life. This was found especially among males as they became more aware of the importance of exercise, medical substances, work-life balance capacity, sleep, and rest to manage obesity. This was further supported by the Global Status Report on Non-communicable Diseases from WHO, which revealed that the prevalence of insufficient physical activity among Malaysians was more apparent in women (58.0%) than in men (46.7%). In addition, perceived stress was identified as a significant predictor of BMI among male respondents as their low tolerance of stress might influence their eating behaviour, either under or overeating [19]. Empirical evidence from previous studies revealed a significant linkage between chronic life stress and obesity [33,62,77,78,79]. The relationship between low tolerance of stress and increased BMI was attributed to neurobiology stress. This leads to hunger and comfort eating that causes a reduction of lipolytic growth hormones, thus leading to fat accumulation [80].

For females, quality of life in the psychological domain was also a significant predictor of BMI. Women may lack a positive outlook of their body image owing to a powerful but false concept of a ‘slim’ body as the only and/or ultimate body shape. Furthermore, a ’thin’ body is perceived as being more socially valued among women in the higher socioeconomic class [81]. Obese females have degrading mental health as they are more concerned with the societal standards of beauty and obesity stigma. In this study, “stressful life events” were a significant predictor of BMI among female respondents. Females are more emotionally vulnerable to social stressors that trigger negative metabolic consequences. As a result, they turn to eat high dietary palatable foods, which, in turn, promote weight gain [82]. Moreover, one possible mechanism that could explain this unhealthy behavioural response is the activation of the pituitary adrenal (HPA) axis, thus increasing the cortisol and leptin levels and central adiposity [82].

This study’s strength is the sampling method used to reduce bias and improve the representativeness of the study sample. Although this study was conducted in only one state (Selangor), to date no other study has examined the association between psychological determinants and obesity in the Malaysian community. Therefore, this study’s outcome can be used as a reference point for future studies involving the general population of Malaysia. The findings of this study can be used to develop more focused community mental health interventions as a strategy for obesity management. The hypothesized relation between psychological determinants and obesity is compounded by diet [83]. Future intervention investigation is needed to establish the mechanisms that link diet and mental well-being to improve weight management. However, the correlation between BMI and body fatness is relatively strong [18]. It overestimates obesity in muscular individuals and underestimates in the elderly who have lost body mass. Besides, this study does not reveal the number of years the respondents had been diagnosed with chronic diseases. Thus, it is difficult to establish the true association between current obesity status and history of chronic diseases. The limitation of the temporal relationship in this study may be that the risk factors and BMI would be measured simultaneously, and one would be hard-pressed to say which came first. Therefore, future studies should include longitudinal designs to better explicate the psychological predictors of obesity in terms of determining causal relationships and direction. It is also important to evaluate the role of confounding variables such as socio-demographic background and history of chronic diseases on psychological risk, especially in different underlying biological pathways in the development of obesity.

## 5. Conclusions

Female adults, aged between 30 to 39 years old, being Indian, married, and having only primary school education or secondary school education were significantly associated with increased risk of obesity. Psychological determinants of obesity for males are perceived stress, suicidal ideation, and quality of life in the physical health domain. Among females, stressful life events and quality of life in the psychological domain contributed significantly to obesity. Nevertheless, this study’s outcomes can be used to integrate psychological approaches to enhance the effectiveness of obesity prevention strategies such as weight-loss programs in community settings. Therefore, it would be beneficial to consider integrating obesity control programs with psychological intervention programs delivered by health professionals to tackle this epidemic that is becoming more prevalent and is placing much stress on the nation’s healthcare resources.

## Figures and Tables

**Table 1:** Characteristics of the respondents in the community of Selangor (*N* = 1380).

Characteristics	Overall (*N* = 1380)	Male (*N* = 513)	Female (*N* = 867)
	*N* (%)/Mean (*SD*)		
Age group (years)			
19 and below	76 (5.5)	41 (8.0)	35 (4.0)
20–29	509 (36.9)	188 (36.6)	321 (37.0)
30–39	351 (25.4)	124 (24.2)	227 (26.2)
40–49	221 (16.0)	72 (14.0)	149 (17.2)
50–59	128 (9.3)	42 (8.2)	86 (9.9)
60 and above	95 (6.9)	46 (9.0)	49 (5.7)
Ethnicity			
Malay	954 (69.1)	360 (70.2)	594 (68.5)
Chinese	112 (8.1)	52 (10.1)	60 (6.9)
Indian	281 (20.4)	87 (17.0)	194 (22.4)
Others	33 (2.4)	14 (2.7)	19 (2.2)
Residence			
Urban	1285 (93.1)	474 (92.4)	811 (93.5)
Rural	95 (6.9)	39 (7.6)	56 (6.5)
Marital status			
Single	472 (34.2)	221 (43.1)	251 (29.0)
Married	851 (61.7)	276 (53.8)	575 (66.3)
Widowed	49 (3.6)	14 (2.7)	35 (4.0)
Divorced	6 (0.4)	2 (0.4)	4 (0.5)
Separated	2 (0.1)	0 (0.0)	2 (0.2)
Education level			
Primary	115 (8.4)	27 (23.5)	88 (76.5)
Secondary	681 (49.9)	258 (37.9)	423 (62.1)
Tertiary	568 (41.6)	224 (39.4)	344 (60.6)
Employment status			
Employed	702 (51.6)	350 (69.0)	352 (41.2)
Unemployed	599 (44.0)	120 (23.7)	479 (56.1)
Retired	60 (4.4)	37 (7.3)	23 (2.7)
History of Medical Problems			
Yes	325 (23.6)	118 (23.0)	207 (23.9)
No	1055 (76.4)	395 (77.0)	660 (76.1)
History of Mental Health Problems			
Yes	22 (1.6)	11 (2.1)	11 (1.3)
No	1358 (98.4)	502 (97.9)	856 (98.7)
History of Mental Health Problems in the Family			
Yes	35 (2.5)	10 (1.9)	25 (2.9)
No	1284 (93.0)	478 (93.2)	806 (93.0)
Not sure	61 (4.4)	25 (4.9)	36 (4.2)
Disability			
Yes	9 (0.7)	5 (1.0)	4 (0.5)
No	1371 (99.3)	508 (99.0)	863 (99.5)
Perceived stress (PSS) (0–40) ± SD	15.08 (4.77)	15.07 (4.70)	15.09 (4.81)
Self-esteem (RSES) (0–40) ± SD	19.65 (3.68)	19.77 (3.66)	19.58 (3.69)
Depression (PHQ-9) ± SD	3.53 (3.58)	3.56 (3.85)	3.52 (3.41)
Present (PHQ-9≥10)	98 (7.1)	46 (9.0)	52 (6.0)
Absent (PHQ-9<10)	1282 (92.9)	467 (91.0)	815 (94.0)
Anxiety (GAD-7) ± SD	1.94 (2.67)	1.80 (2.66)	2.03 (2.68)
Present (GAD-7 ≥ 8)	69 (5.0)	25 (4.9)	44 (5.1)
Absent (GAD-7 < 8)	1311 (95.0)	488 (95.1)	823 (94.9)
Suicidal Ideation			
Yes	97 (7.0)	49 (9.6)	48 (5.5)
No	1283 (93.0)	464 (90.4)	819 (94.5)
Stressful Life Event ± SD	3.56 (1.74)	3.59 (1.84)	3.54 (1.67)
No	37 (2.7)	9 (1.8)	28 (3.2)
Low (1 stressful life event)	152 (11.0)	68 (13.3)	84 (9.7)
Moderate (2 stressful life events)	182 (13.2)	78 (15.2)	104 (12.0)
High (≥3 stressful life events)	1009 (73.1)	358 (69.8)	651 (75.1)
Quality of Life (WHOQOL-BREF-26) ± SD			
Physical Health (4–20)	14.79 (2.16)	14.84 (2.12)	14.76 (2.18)
Psychological Health (4–20)	14.56 (2.09)	14.49 (2.20)	14.60 (2.03)
Social Relationships (4–20)	15.28 (2.42)	15.38 (2.50)	15.22 (2.38)
Environment (4–20)	14.66 (1.97)	14.59 (2.04)	14.70 (1.93)
Weight (kg) Mean ± SD	65.77 (15.42)	70.85 (14.81)	62.77 (15.00)
Height (cm) Mean ± SD	161.25 (9.39)	167.60 (8.97)	157.49 (7.40)
Body mass index (kg/m^2^) Mean ± SD	25.34 (5.79)	25.26 (5.14)	25.38 (6.15)

Maximum Perceived Stress Scale (PSS) score = 40. Higher scores indicate higher perceived stress. Maximum Rosenberg’s Self-esteem Scale (RSES) score = 40. Higher scores indicate higher self-esteem. Maximum physical health domain score = 20. Higher scores indicate higher quality of life in physical health domain. Maximum psychological domain score = 20. Higher scores indicate higher quality of life in psychological domain. Maximum social relationships domain score = 20. Higher scores indicate higher quality of life in social relationships domain. Maximum environment domain score = 20. Higher scores indicate higher quality of life in the environment domain.

**Table 2:** Prevalence of obesity by socio-demographic characteristics among respondents (*N* = 1380).

Characteristics	Obesity *N* (%)		
Overall (*N* = 1380)	256 (18.6)		
Male (*N* = 513)	73 (14.2)		
Female (*N* = 867)	183 (21.1)		
	Male	Female	Overall
Age group (years)			
19 and below	7 (17.1)	3 (8.6)	10 (13.2)
20–29	30 (16.0)	44 (13.7)	74 (14.5)
30–39	15 (12.1)	64 (28.2)	79 (22.5)
40–49	10 (13.9)	39 (26.2)	49 (22.2)
50–59	4 (9.5)	20 (23.3)	24 (18.8)
60 and above	7 (15.2)	13 (26.5)	20 (21.1)
Ethnicity			
Malay	50 (13.9)	120 (20.2)	170 (17.8)
Chinese	5 (9.6)	11 (18.3)	16 (14.3)
Indian	18 (20.7)	50 (25.8)	68 (24.2)
Other	0 (0.0)	2 (10.5)	2 (6.1)
Residence			
Urban	70 (14.8)	170 (21.0)	240 (18.7)
Rural	3 (7.7)	13 (23.2)	16 (16.8)
Marital status			
Single	38 (17.2)	29 (11.6)	67 (14.2)
Married	33 (12.0)	140 (24.3)	173 (20.3)
Widow/widower	2 (14.3)	11 (31.4)	13 (26.5)
Divorced	0 (0.0)	1 (25.0)	1 (16.7)
Separated	0 (0.0)	2 (100.0)	2 (100.0)
Education level			
Primary	3 (11.1)	29 (33.3)	32 (27.8)
Secondary	33 (12.8)	109 (25.8)	142 (20.9)
Tertiary	36 (16.1)	42 (12.2)	78 (13.7)
Employment status			
Employed	51 (14.6)	72 (20.5)	123 (17.5)
Unemployed	17 (14.2)	104 (21.7)	121 (20.2)
Retired	4 (10.8)	4 (17.4)	8 (13.3)

**Table 3:** Association between obesity and socio-demographic characteristics among respondents (*N* = 1380).

Characteristics	Obese	Non-Obese	Chi-Square	Crude OR (95%CI)	*p* Value
Gender			10.09		0.001 *(0.086)
Female	183 (21.1%)	684 (78.9%)		1.61(1.20–2.17)	
Male	73 (14.2%)	440 (85.8%)			
Age (years)			12.84		0.025 * (0.096)
Below 19	10 (13.2%)	66 (86.8%)		0.65 (0.33–1.29)	0.213
20–29	74 (14.5%)	435 (85.5%)		0.64 (0.48–0.87)	0.003 *
30–39	79 (22.5%)	272 (77.5%)		1.40 (1.04–1.88)	0.027 *
40–49	49 (22.2%)	172 (77.8%)		1.31 (0.92–1.86)	0.131
50–59	24 (18.8%)	104 (81.3%)		1.02 (0.64–1.62))	0.951
60 and above	20 (21.1%)	75 (78.9%)		1.19 (0.71–1.98)	0.516
Ethnicity			11.03		0.012 * (0.089)
Malay	170 (17.8%)	784 (82.2%)		0.86 (0.64–1.14)	0.296
Chinese	16 (14.3%)	96 (85.7%)		0.71 (0.41–1.23)	0.226
Indian	68 (24.2%)	213 (75.8%)		1.55 (1.13–2.12)	0.006 *
Others	2 (6.1%)	31 (93.9%)		0.28 (0.07–1.17)	0.062
Residence			0.20	1.13 (0.65–1.98)	0.657
Urban	240 (18.7%)	1045 (81.3%)			
Rural	16 (16.8%)	79 (83.2%)			
Marital status			18.57		0.001 * (0.116)
Single	67 (14.2%)	405 (85.8%)		0.63 (0.46–0.85)	0.003 *
Married	173 (20.3%)	678 (79.7%)		1.37 (1.03–1.83)	0.031 *
Widowed	13 (26.5%)	36 (73.5%)		1.62 (0.85–3.10)	0.143
Divorced	1 (16.7%)	5 (83.3%)		0.88 (0.10–7.55)	0.691 ^a^
Separated	2 (100.0%)	0 (0.0%)		-	0.034 ^a,^*
Education level			17.71		<0.001 * (0.114)
Primary	32 (27.8%)	83 (72.2%)		1.80 (1.17–2.78)	0.007 *
Secondary	142 (20.9%)	539 (79.1%)		1.37 (1.04–1.81)	0.024 *
Tertiary	78 (13.7%)	490 (86.3%)		0.57 (0.43–0.77)	<0.001 *
Employment status			2.66		0.256
Employed	123 (17.5%)	579 (82.5%)		0.87 (0.66–1.15)	0.330
Unemployed	121 (20.2%)	478 (79.8%)		1.22 (0.93–1.60)	0.156
Retired	8 (13.3%)	52 (86.7%)		0.67 (0.31–1.42)	0.290

* significant at *p* < 0.05. () is the measure of significant association (2 × 2 for phi coefficient and others for contingency coefficient), using Guildford’s rule of thumb to interpret the magnitude of association between the two variables. ^a^ = Fisher’s Exact Test.

**Table 4:** Association between obesity and chronic diseases among respondents (*N* = 1380).

Characteristics	Obese	Non-Obese	Chi-Square	Crude OR (95%CI)	*p* Value
Chronic disease			19.00		<0.001 * (0.117)
Yes	87 (26.8%)	238 (73.2%)		1.92 (1.43–2.56)	
No	169 (16.0%)	886 (84.0%)			
Heart disease			0.25		0.620
Yes	6 (22.2%)	21 (77.8%)		1.26 (0.50–3.16)	
No	250 (18.5%)	1103 (81.5%)			
Diabetes			10.37		0.001 * (0.087)
Yes	33 (30.0%)	77 (70.0%)		2.01 (1.31–3.10)	
No	223 (17.6%)	1047 (82.4%)			
Stroke			0.20		0.546 ^a^
Yes	1 (12.5%)	7 (87.5%)		0.63 (0.08–5.11)	
No	255 (18.6%)	1117 (81.4%)			
Hypertension			22.80		<0.001 * (0.129)
Yes	52 (32.3%)	109 (67.7%)		2.37 (1.65–3.41)	
No	204 (16.7%)	1015 (83.3%)			
Arthritis			2.72		0.099
Yes	10 (29.4%)	24 (70.6%)		1.86 (0.88–3.95)	
No	246 (18.3%)	1100 (81.7%)			
Cancer			0.69		0.408 ^a^
Yes	0 (0.0%)	3 (100.0%)		-	
No	256 (18.6%)	1121 (81.4%)			
Asthma			3.99		0.046 * (0.054)
Yes	20 (27.4%)	53 (72.6%)		1.71 (1.00–2.92)	
No	236 (18.1%)	1071 (81.9%)			
Kidney Failure			0.56		0.499
Yes	0 (0.0%)	2 (100.0%)			
No	256 (18.6%)	1122 (81.4%)			
Thyroidism			5.27		0.043 ^a,^* (0.062)
Yes	4 (50.0%)	4 (50.0%)		4.44 (1.10–17.89)	
No	252 (18.4%)	1120 (81.6%)			

* significant at *p* < 0.05. () is the measure of significant association (2 × 2 for phi coefficient and others for contingency coefficient), using Guildford’s rule of thumb to interpret the magnitude of association between the two variables. ^a^ = Fisher’s Exact Test.

**Table 5:** Relationship of Perceived stress (PSS), Self-esteem (RSES), Depression (PHQ-9), Anxiety (GAD-7), Suicidal Ideation, Stressful Life Event, QOL—Physical Health, QOL—Psychological Health, QOL—Social Relationships and QOL—Environment to body mass index (BMI)—Male Respondents.

Psychological Variables	Simple Linear Regression			Multiple Linear Regression		
	B	t	*p*	B	t	*p*
Perceived stress (PSS) (0–40)	−0.075	−1.559	0.120	−0.107	−2.046	0.041 *
Self-esteem (RSES) (0–40)	0.077	1.235	0.217			
Depression (PHQ-9)	−0.090	−1.526	0.128			
Anxiety (GAD-7)	0.050	0.584	0.559			
Suicidal Ideation	−2.062	−2.686	0.007	−2.423	−3.031	0.003 *
Stressful Life Event	0.088	0.712	0.477			
Quality of Life (WHOQOL-BREF-26)						
QOL—Physical Health (4–20)	−0.158	−1.470	0.142	−0.350	−2.945	0.003 *
QOL—Psychological Health (4–20)	−0.167	−1.621	0.106			
QOL—Social Relationships (4–20)	−0.012	−0.135	0.893			
QOL—Environment (4–20)	0.050	0.451	0.652			

* significant with 2-tailed test. Variables with a *p* value of less than 0.25 in simple linear regression were included in the multiple linear regression model. Adjusted R Square = 0.03, F = 5.69; *p* = 0.001.

**Table 6:** Relationship of Perceived stress (PSS), Self-esteem (RSES), Depression (PHQ-9), Anxiety (GAD-7), Suicidal Ideation, Stressful Life Event, QOL—Physical Health, QOL—Psychological Health, QOL—Social Relationships and QOL—Environment to body mass index (BMI)—Female Respondents.

Psychological Variables	Simple Linear Regression			Multiple Linear Regression		
	B	t	*p*	B	t	*p*
Perceived stress (PSS) (0–40)	0.017	0.403	0.687			
Self-esteem (RSES) (0–40)	0.122	2.167	0.031			
Depression (PHQ-9)	0.055	0.899	0.369			
Anxiety (GAD-7)	−0.004	−0.049	0.961			
Suicidal Ideation	0.043	0.047	0.962			
Stressful Life Event	0.703	5.730	<0.001	0.711	5.869	<0.001 *
Quality of Life (WHOQOL-BREF-26)						
QOL—Physical Health (4–20)	−0.427	−4.511	<0.001			
QOL—Psychological Health (4–20)	−0.469	−4.618	<0.001	−0.478	−4.789	<0.001 *
QOL—Social Relationships (4–20)	−0.207	−2.363	0.018			
QOL—Environment (4–20)	−0.195	−1.807	0.071			

* significant with 2-tailed test. Variables with a *p* value of less than 0.25 in simple linear regression were included in the multiple linear regression model Adjusted R Square = 0.06, F = 28.30; *p* < 0.001.

## Data Availability

The data presented in this study are available on request from the corresponding author.
